# Expression and significance of α-SMA and PCNA in the vascular adventitia of balloon-injured rat aorta

**DOI:** 10.3892/etm.2013.1059

**Published:** 2013-04-09

**Authors:** XIANGJUN WU, QINGHUA LU

**Affiliations:** 1 Shandong University, Jinan, Shandong 250000, P.R. China; 2 Department of Cardiology, The Second Hospital of Shandong University, Jinan, Shandong 250000, P.R. China

**Keywords:** α-smooth muscle actin, proliferating cell nuclear antigen, adventitial cells, restenosis

## Abstract

The aim of this study was to investigate changes in the expression of α-smooth muscle actin (α-SMA) and proliferating cell nuclear antigen (PCNA) in the vascular adventitia of balloon-injured rat aortas in the second and sixth postoperative weeks. A total of 32 rats were divided into a control group and a balloon-injured group. The rats underwent vascular morphometric analysis and adventitial cell counting, as well as immunohistochemical staining of α-SMA and PCNA in postoperative weeks 2 and 6 for observation of the expression of each immune parameter in the vascular adventitia and calculation of the number of PCNA-positive nuclei and the PCNA labeling index (PCNALI) in the vascular adventitia. The area and thickness of the adventitia, the number of nuclei and the PCNALI of the vascular adventitia were significantly increased in the injured group compared with the control group (P<0.05), while the external elastic lamina area (EELA), internal elastic lamina area (IELA) and lumen area (LA) were significantly decreased (P<0.05) in the second week. The area and thickness of the adventitia, the number of nuclei and the PCNALI of the vascular adventitia were significantly increased in the injured group compared with the control group (P<0.05), while the EELA, IELA and LA were significantly reduced (P<0.05) in the sixth week, and were significantly lower than those in the injured group in the second week (P<0.05). The positive expression levels of α-SMA and PCNA in the vascular adventitia were significantly reduced compared with those in the second week after injury. The vascular adventitial cells underwent proliferation and phenotypic switching and participated in vascular remodeling and vascular restenosis following balloon-induced injury. The vascular contractile remodeling in the injured group was more evident in the sixth week than in the second week, followed by a more aggravated vascular stenosis. Consequently, the vascular remodeling was one of the causes of vascular restenosis.

## Introduction

Coronary heart disease is one of the most common and serious diseases affecting human health. Since Gruentzig (1) completed the first percutaneous transluminal coronary angioplasty (PTCA) in Zurich, Switzerland in 1977, interventional therapy has become an effective means of treating coronary heart diseases; however, PTCA has a high incidence of restenosis (2). Coronary stent implantation effectively suppresses the vascular elastic recoil, negative remodeling and thrombosis; however, 15–30% cases present in-stent restenosis (3). The higher incidence of restenosis increases patients’ suffering and the economic burden imposed on society, and affects the long-term efficacy and clinical application of PTCA. Research into the mechanisms of restenosis to assist in the effective prevention of restenosis is the key to improving the effects of percutaneous coronary intervention, which is an issue of serious concern to cardiovascular physicians.

The mechanisms of restenosis following PTCA involve the synthesis, release and chemical chemotaxis of inflammatory cytokines and growth factors, the degradation and synthesis of extracellular matrix (ECM), phenotypic switching, proliferation and migration of smooth muscle cells, neointimal thickening and vascular remodeling (VR). Using histology and microscopy, studies have focused on the medial membrane and intima of the arteries (4). However, few studies have considered the vascular adventitia. The role of the vascular adventitia is gradually gaining attention (5). Shi *et al* (6) reported that the adventitia of pig coronary arteries change following balloon injury. A study of restenosis following angioplasty demonstrated that in addition to the smooth muscle cells of the vascular intima, the vascular adventitial fibroblasts were also involved in restenosis (7), indicating that the adventitia is not only a ‘bystander’ in a traditional sense, but also combines with the smooth muscle cells of the intima and medial membrane to participate in the restenosis of blood vessels. However, the mechanism of the vascular adventitia in restenosis following angioplasty remains unclear. This study aimed to explore the mechanism of the vascular adventitia, particularly the role of fibroblasts, in vascular restenosis following balloon injuries. Pure line Sprague-Dawley (SD) rats were used to establish the restenosis models following angioplasty based on balloon-induced intimal injuries. The rats underwent pathological hematoxylin and eosin (H&E) staining and immunohistochemical staining of the injured blood vessels and image analysis processing in the second and sixth postoperative week to explore the VR in the restenosis models of injured blood vessels, as well as the mechanisms of activation, proliferation and phenotypic switching in vascular adventitia cells. The effect and mechanism of the vascular adventitia in vascular restenosis following balloon-induced intimal injuries were observed based on whole-animal experiments, and may lead to a new approach in the prevention and treatment of vascular restenosis.

## Materials and methods

### Animal groups

A total of 32 healthy female SD rats weighing 200±20 g were provided by the Experimental Animal Research Center of Ruijin Hospital affiliated to Shanghai Jiaotong University. The 6-week-old pure line SD rats were divided into two groups, including the control group (n=16) and the balloon-injured group (n=16). This study was performed in strict accordance with the recommendations in the Guide for the Care and Use of Laboratory Animals of the National Institutes of Health. The animal use protocol was reviewed and approved by the Institutional Animal Care and Use Committee (IACUC) of Shandong University.

### Establishment of animal models

The rats in the balloon-injured group underwent intraperitoneal anesthesia with ketamine and were fixed in the supine position. A midline incision of the abdomen was made under sterile conditions, through which the small intestine and part of the large intestine were removed and wrapped with physiological saline gauzes. The abdominal aorta and inferior vena cava were bluntly dissected and a transverse incision was made ∼1 cm below the renal artery branch of the abdominal aorta. A balloon catheter was inserted retrogradely along the abdominal aorta into the aortic arch, to a depth of ∼7–8 cm. The balloon was filled with 8–10 atm heparin saline solution and then slowly pulled back to the incision, reducing the pressure inside the balloon at the same time to maintain a certain resistance. Thereafter, the balloon catheter was inserted into the aortic arch again and then was finally withdrawn after being repeated three times. Following balloon injury surgery, the incision and intravascular thromboses were washed with heparin saline solution. The incision was sutured using 4–5 needles with an 8/0 vascular suture under a surgical microscope. Before the last two stitches of suturing, intravascular bubbles were discharged with heparin saline solution. The rats underwent postoperative intraperitoneal injection of 800,000 U/kg body weight penicillin for 3 days.

### Production of paraffin sections

Each group of rats was randomly narcotized and sacrificed (n=16) on the second and sixth postoperative weeks. Their aortas from the aortic arches to the horizontal segments of the abdominal aorta in the openings of the renal arteries were immediately removed and washed with physiological saline to remove the attached blood cells. The abdominal aortic segments were clipped with surgical scissors and fixed in 4% neutral paraformaldehyde to prepare the paraffin sections.

### Immunohistochemical detection and histomorphological analysis

The vascular ring of the paraffin sections underwent immunohistochemical staining using the SABC method. Firstly, mouse anti-rat proliferating cell nuclear antigen (PCNA; Zymed Lab Inc., San Diego, CA, USA) and α-smooth muscle actin (α-SMA; Neomarkers Inc., Fremont, CA, USA) antibodies were added (1:200 dilution) and the sections were incubated overnight at 4°C, with phosphate-buffered saline (PBS) as the negative control. Then, the goat anti-rabbit (Wuhan Boster Biological Technology Ltd., Hubei, China) and the goat anti-mouse secondary antibodies (Fuzhou Maixin Biological Technology Co., Fujian, China) were added and the sections were incubated for 30 min at 37°C, followed by washing with PBS and 3,3′-diaminobenzidine (DAB) chromogenesis. The expression levels of PCNA and α-SMA in the vascular adventitia, medial membrane and intima were observed in each group. The paraffin sections underwent H&E staining and measurement of the lumen area (LA), external elastic lamina area (EELA), internal elastic lamina area (IELA) and the area of vascular adventitia and surrounding adipose tissue at each time point using a Leica software image analysis system (Leica, Munich, Germany). VR was evaluated according to the changes of vascular remodeling index (VRI), IELA and EELA, while vascular stenosis was evaluated according to the changes in residual stenosis rate and vessel LA.

### Statistical analysis

SPSS v11.5 software (SPSS, Inc., Chicago, IL, USA) was used for statistical analysis. Measurement data are presented as mean ± standard deviation. Comparisons between the two groups were performed using t-test and inter-group comparisons using analysis of variance. P<0.05 was considered to indicate a statistically significant difference.

## Results

### Establishment of the restenosis model following balloon injury

Among the 34 rats, 2 rats died due to excessive bleeding during balloon injury of the endothelium. Therefore, 32 rats were included in the study.

### Results of H&E staining

**Figure 1:**
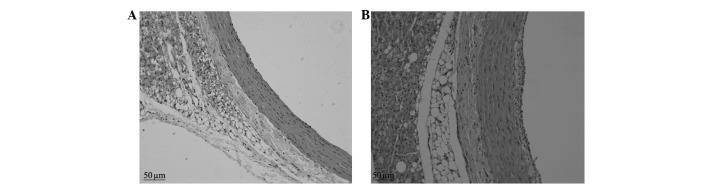
H&E staining on the second week after balloon injury (magnification, ×200). (A) Control group. (B) Injury group demonstrated vascular adventitial thickening, neointimal formation and contractile remodeling of blood vessels. H&E, hematoxylin and eosin.

**Figure 2:**
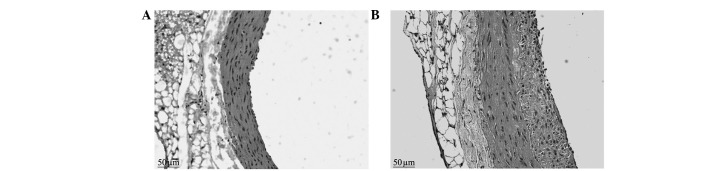
H&E staining on the sixth week after balloon injury (magnification, ×200). (A) Control group. (B) Injury group demonstrated a large number of neointimal formation and contractile remodeling of blood vessels, as well as aggravated vascular stenosis compared with the second week group. H&E, hematoxylin and eosin.

The control group presented vascular walls of even thickness, a single layer of endothelial cells in the vessel lumen surface and large round nuclei which were stained blue-violet. There were no new smooth muscle cells visible in the intima; however, there was a clear and complete undulating internal and external elastic lamina and the intima comprised a single layer of endothelial cells and an extremely small amount of ECM. There were 6–10 layers of wavy elastic fibers visible in the even thickness of the medial membrane, between which smooth muscle cells were visible, with fusiform or almost round nuclei of approximately the same size. The adventitia was located between the external elastic lamina and the perivascular adipose tissue, constituted by loose connective tissue, including helical or longitudinal distribution of elastic fibers, collagen fibers and fibroblasts (Figs. 1 and 2).

The neointimal formation was visible under a microscope in the balloon-injured group in the second postoperative week. The LA was reduced compared with that of the control group and the internal elastic lamina presented multiple fractures, the vascular adventitia was thickened compared with that of the control group and the number of nuclei was increased. In the sixth week, a large amount of neointima was visible on the intimal surface of injured blood vessels under a microscope. The proliferous neointima had an extremely uneven thickness, uneven vascular walls and irregular vessel lumen surfaces. The vessel LAs were clearly reduced compared with those of the control group. There was abundant cell proliferation in the neointima, with a clear increase in the number of blue nuclei, which were disordered and arranged in different directions. The nuclei varied in size and shape, and multiple fractures were visible in the internal elastic lamina. The medial membrane was of uneven thickness and there was no clear change in the cell morphology. The vascular adventitia was thicker than that of the control group, with an increased number of nuclei and thickened vascular walls (Figs. 1 and 2).

### Histomorphological analysis

A Leica image analysis system was used to measure the area and thickness of the vascular adventitia in the two groups, as well as the number of nuclei following H&E staining. The results revealed that in the second postoperative week: the area of the vascular adventitia (0.389±0.048 vs. 0.255±0.030 mm^2^; P<0.05), the vascular adventitia thickness (0.152±0.023 vs. 0.096±0.011 mm; P<0.05), and the number of nuclei (28.63±3.50 vs. 22.25±3.69; P<0.05) were increased compared with those of control group. In the sixth postoperative week: the area of the vascular adventitia (0.337±0.066 vs. 0.255±0.029 mm^2^; P<0.05); the vascular adventitia thickness (0.130±0.014 vs. 0.095±0.013 mm; P<0.05); and the number of nuclei (44.88±5.62 vs. 15.75±3.50; P<0.05) were also increased compared with those of the control group.

### Changes in vessel LA, IELA, EELA and VR

The LA, IELA, EELA, VRI and the residual stenosis rate of the two groups were measured on the second and sixth weeks after balloon injury. The results revealed that in the injury group in the second week compared with the control group in the second and sixth weeks: the LA was significantly reduced (1.299±0.011 vs. 1.592±0.046 and 1.608±0.001 mm^2^, respectively; P<0.05); the IELA was significantly reduced (1.349±0.109 vs. 1.592±0.046 and 1.608±0.001 mm^2^, respectively; P<0.05); and the EELA was significantly reduced (1.740±0.106 vs. 2.012±0.037 and 2.005±0.047 mm^2^, respectively; P<0.05). In the injured group in the sixth week compared with the control group in the second and sixth weeks: the LA was significantly reduced (1.101±0.007 vs. 1.592±0.046 and 1.608±0.001 mm^2^, respectively; P<0.05); the IELA was significantly reduced (1.199±0.003 vs. 1.592±0.046 and 1.608±0.001 mm^2^, respectively; P<0.05); and the EELA was significantly reduced (1.504±0.001 vs. 2.012±0.037 and 2.005±0.047 mm^2^, respectively; P<0.05). The intimal area in the injured group in the sixth week was significantly thickened compared with that of the injured group in the second week (0.099±0.007 vs. 0.050±0.011 mm^2^; P<0.05).

### Immunohistochemical staining

**Figure 3:**
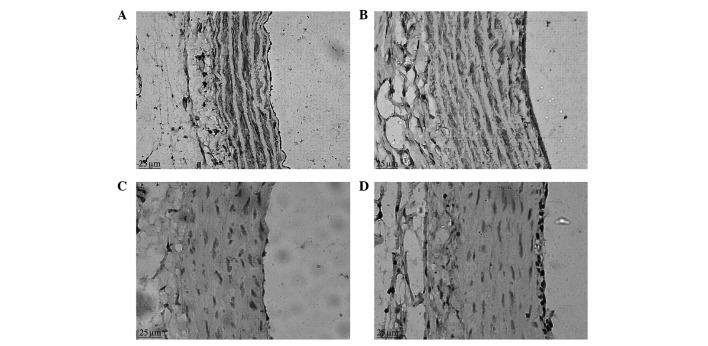
Expressions of α-SMA and PCNA in the second week after balloon injury (magnification, ×400). α-SMA staining in (A) the control group and (B) the injury group; PCNA staining in (C) the control group and (D) PCNA the injury group. SMA, smooth muscle actin; PCNA, proliferating cell nuclear antigen.

In the control group, when observed under a light microscope, a weak positive expression of α-SMA was observed in the vascular adventitia; a strong brown positive expression of α-SMA was clearly visible in the vascular medial membrane, evenly distributed in the cytoplasm of vascular smooth muscle cells (VSMCs), with blue nuclei, and there was no positive expression of α-SMA in the vascular intima. In the balloon-injured group in the second week, a positive expression of α-SMA was visible in the vascular adventitia; there was a stronger positive expression of α-SMA in the neointimal layer and a lower level of expression in the medial membrane, with variable intensity and uneven distribution, significantly reduced compared with that of the normal control group (Fig. 3). In the balloon-injured group in the sixth week, the expression of α-SMA in the vascular adventitia and medial membrane was similar to that of the control group and positive expression of α-SMA was visible in the thicker intima.

The positive expression of PCNA was observed to be located in the nucleus, the positive staining was brown and the negative staining was blue. Under a light microscope, brown positive expression indicative of PCNA was occasionally observed in the vascular adventitia, medial membrane and intima in the control group. In the balloon-injured group in the second week, there was a large amount of strong brown positive expression of PCNA visible in the vascular adventitia; there was also strong positive expression of PCNA visible in the neointima, which tended to be aggregated at the luminal surface (Fig. 3). In the balloon-injured group in the sixth week, the vascular adventitia cells were fewer in number than in the second week; the proliferating cells with brown nuclei were hardly visible; proliferating cells were occasionally visible in the medial membrane; and there was positive expression of PCNA visible in the thicker neointima. There was a statistical difference in the vascular proliferation index between the balloon-injured and control groups (22.59±5.29 vs. 6.90±1.10; P<0.05).

## Discussion

Glagov *et al* (8) identified that with the growth of intracoronary atherosclerotic plaques in humans, the lumen expands in a compensatory effect. This has also been observed in a large number of animal experiments and autopsies (9). As research has progressed, the concept of VR has been proposed and it is considered that VR may play an important role in lumen stenosis. VR is a chronic change in the vascular diameter or a structural change in the vascular wall; therefore, it changes the ratio of the size of the vascular lumen to the vascular wall or the geometrical shape, including an increase or reduction of the vascular cross-sectional area. VR is associated with corresponding adjustments to hemodynamic functions and vascular wall structures under the conditions of continuous adaptation to different physiological and pathological states, which are the pathophysiological basis of vascular diseases. Remodeling processes include the expansionary remodeling of blood vessels (also known as positive remodeling, outward remodeling or compensatory remodeling) with a VRI >1 and contractile remodeling (also known as negative remodeling, inward remodeling or decompensatory remodeling) with a VRI <1. Expansionary remodeling may prevent the vascular lumen from stenosis through vascular compensatory expansion, while contractible remodeling may cause inadequate compensation due to plaque formation or vasoconstriction, resulting in vascular lumen stenosis (10).

A quantitative histological method is the most common method used to evaluate VR. In the current study, multiple parameters of morphological changes in rat VR following balloon injury were quantitatively analyzed using an image analysis system. VR was evaluated according to the changes of VRI, IELA and EELA, while vascular stenosis was evaluated according to the changes of residual stenosis rate and vessel LA. In this study, compared with the control group, the injured group in the second week presented clear retraction of the blood vessels and a reduction in the area of the vascular lumen (P<0.05), as well as reduced EELA and IELA of blood vessels (P<0.05). The area of the neointima was 0.050±0.011 mm^2^. The VRI was 0.865, indicating contractile remodeling. The injured group in the sixth week presented a greater reduction in the area of the vascular lumen (P<0.05) and significantly reduced EELA and IELA of blood vessels (P<0.05) compared with that in the second week. The area of the neointima increased to 0.099±0.007 mm^2^. The VRI was 0.750, indicating a further role of the VR in the vascular lumina narrowing process. Vascular contractile remodeling may cause the vascular walls to lose their compensatory abilities to undergo retraction, indicating that the role of contractile remodeling in the vascular luminal narrowing process should not be ignored. One study demonstrated that the main factors affecting the late changes in vascular LA and causing vascular contractile remodeling and restenosis are the imbalance of synthesis and degradation of ECM collagen, as well as the anomalous collagen structure and the fibrosis of the vascular adventitia (11).

In this study, in the blood vessels of the balloon-injured group in the second week compared with those of the control group, the adventitia demonstrated not only a marked increase in the number of cells (P<0.05), but the thickness and area were also significantly increased (P<0.01). There was neointimal hyperplasia; the thickness and area of the adventitia and the number of cells in the balloon-injured group in the sixth week remained elevated compared with those of the control group (P<0.05). There was more apparent neointimal hyperplasia in the lumen in the sixth week than in the the second week. This indicates that when the artery is injured, cell proliferation occurs in the adventitia, leading to thickening in the intima and loss of the vicarious expansion ability, which is a significant cause of vascular restenosis. In this study, the thickness and area of the vascular adventitia gradually increased following balloon injury to the blood vessels, as well as the number of the adventitial cells and the thickness of the intima, which were more evident in the sixth week than the second week. Shi *et al* (12) identified that in a pig model of vascular restenosis following coronary artery injury, the change in vascular adventitial thickness was positively correlated with the change in cell number, which was consistent with our experimental result. This phenomenon may indicate a change in the structure of the vascular adventitia (including connective tissue and fibroblasts), which may be involved in a change in the characteristics of cell phenotype or function. We consider that it may be caused by cell aggregation induced by the phenotypic change and enhanced proliferation ability of the adventitial cells. It may also be related to the deposition of ECM caused by increased collagen synthesized by the adventitial fibroblasts. The proliferation, synthesis and secretion of ECM in the muscle fiber cells of the vascular adventitia may cause the injured arteries to undergo adventitial cicatrization and elastic rebound, causing vascular adventitial remodeling, which is an important factor of vascular restenosis following balloon injury.

Fibroblasts are the main cell type in the vascular adventitia in mammals and humans. It is considered that following arterial injury, fibroblasts participate in vascular repair. In the early stages of balloon injury, the proliferating cells in the vascular adventitia and on the medial membrane are in fusiform hypertrophy, similar to VSMCs but differing from fibroblasts in function (13). Certain scholars consider that these non-muscular cells in the adventitia are derived from fibroblasts; therefore, they are called myofibroblasts (MFs), which synthesize and secrete α-actin (14). This protein was previously considered a specific expressive substance of muscular cells; therefore, the fusiform cells that synthesize and secrete α-actin are called muscle cells (MCs) here. Therefore, myofibroblast is another name for VSMC in a different environment (15). In the present study, the vascular adventitia presented actin protein expression according to immunohistochemical analysis. We observed under a light microscope that in the control group: there was weak positive expression of α-SMA in the vascular adventitia; there was strong brown positive expression of α-SMA clearly visible in the vascular medial membrane; and there was no positive expression of α-SMA in the vascular intima. In the balloon-injured group in the second week: there was positive expression of α-SMA visible in the vascular adventitia; there was a lower level of expression in the medial membrane, with variable intensity and uneven distribution, significantly reduced compared with that of the normal control group; and there was stronger positive expression of α-SMA in the neointimal layer. In the balloon-injured group in the sixth week, the expression of α-SMA in the vascular adventitia and medial membrane was similar to that in the control group and positive expression of α-SMA was visible in the thicker intima. Following balloon injury in the blood vessels, the adventitial fibroblasts underwent phenotypic changes to form MFs, fibroblasts with characteristics of muscle fiber cells, which also secrete ECM and numerous types of biologically active factors to participate in tissue repair. They also have a strong migration ability (migrating to the medial membrane and intima through the lacerated external elastic lamina), as well as synthesis, secretion and shrinkage properties, thus causing restenosis in injured blood vessels. However, further investigation of the expression of desmin and myosin is required to determine the properties of MFs.

A previous study has demonstrated that the proliferation of VSMCs is a characteristic change associated with vascular restenosis formation following balloon injury (16). Therefore, it is important to look for a simple, sensitive and specific evaluation index of cell proliferation. PCNA, a 36 kD nucleoprotein, is an auxiliary DNA polymerase-δ protein in mammals. When resting cells begin to proliferate, the synthesis of PCNA is activated and significantly increased, which is an important biological indicator of proliferation of responding cells (17). Additionally, immunohistochemical staining of PCNA is more sensitive than labeling with ^3^H-thymine or bromodeoxyuridine, the latter of which only measures the cells in the S phase while PCNA also exists in G1 and G2. PCNA has been used in clinical and basic studies of vascular restenosis following balloon injury (18), since its analysis is simple and sensitive. PCNA detection is a relatively reliable index for evaluating the state of cell proliferation (19), since it objectively reflects the activity and distribution of cell proliferation.

Using immunohistochemistry, we identified that there was a large amount of positive expression of PCNA in the vascular adventitia in the balloon-injured group in the second week compared with the control group; the expression levels of PCNA in the vascular adventitia and the medial membrane in the balloon-injured group in the sixth week were similar to those in the control group and there was positive expression of PCNA visible in the thicker neointima. This indicates that the adventitial fibroblasts were in an active state of proliferation and migrated to the intima to form the neointima, which is a similar result to that reported in previous studies (20,21). One study demonstrated that the protein expression of PCNA is closely related to intimal hyperplasia and that the expression level is proportional to the degree of cell proliferation (22).

We identified that the proliferation index of PCNA in the balloon-injured group in the second week was significantly increased compared with that of the control group, with a statistically significant difference, indicating that the number of adventitial cells was increased. Fibroblast proliferation, an early response to vascular injury, relies on reactive oxygen species (ROS), particularly the H_2_O_2_ effect derived from reduced nicotinamide adenine dinucleotide phosphate oxidase II (NADPH). In an animal model of arterial injury, fibroblasts labeled with 5-bromo-2 deoxyuridine (BrdU) were mainly concentrated in the vascular adventitia outside the vascular external elastic layer. Following vascular injury, intima was ischemic and hypoxic. The vascular adventitial fibroblasts quickly adapt to the requirements of the local blood vessels, initiate Gq protein signal transduction pathways, activate protein kinase C (PKC) and promote mitogen activated protein kinase (MAPK) family members, leading to extensive fibroblast proliferation (23). The proliferated vascular adventitial cells have a similar function to muscle cells, which migrate to the intima through the internal and external elastic lamina to constitute the cell components of the new intima. There was also strong positive expression of PCNA visible in the neointima in the vascular injury group, which tended to be aggregated at the lumen surface. Therefore, the proliferation of adventitial cells was confirmed to be one of the causes of vascular restenosis, consistent with the conclusion of Diez-Juan and Andrés (24).

In conclusion, following vascular injury, the adventitia area was increased. At the same time, EELA, IELA and LA were reduced, with a remodeling index <1 and contractile remodeling in the vessels, which was more apparent in the sixth postoperative week compared with the second postoperative week. The neointimal area was gradually increased as the LA reduced, indicating aggravated vascular stenosis. Therefore, in the different periods following balloon injury, the changes in the expression of α-SMA and PCNA in the vascular adventitia demonstrated that the vascular adventitial cells were activated to cause proliferation and phenotypic switching, participating in VR and vascular restenosis following balloon injury. Fully understanding and using this mechanism may provide new ideas for the future development of new drugs for the treatment of cardiovascular diseases. This study requires further investigation with regard to the adventitial cell factor activation signal pathway, with a larger number of cases.

## References

[1] Gruentzig AK (1978). Transluminal dilatation of coronary-artery stenosis. Lancet.

[2] Gasterell PJ, Teirstein PS (1999). Prevention of coronary restenosis. Cardio Rev.

[3] Smith SC, Dove JT, Jacobs AK (2001). ACC/AHA guidelines of percutaneous coronary intervention (revision of the 1993 PTCA guidelines)-executive summary. A report of the American College of Cardiology/American Heart Association Task Force on Practice Guidelines (committee to revise the 1993 guidelines for percutaneous transluminal coronary angioplasty). J Am Coll Cardiol.

[4] Raines EW (2000). The extracellular matrix can regulate vascular cell migration, Proliferation and survival: relationships to vascular disease. Int J Exp Pathol.

[5] Shen K, Liu FJ, Yuan GY (2010). Effect of paclitaxel on the expression of vascular fibroblast of matrix metalloproteinases-2 and α-smooth muscle actin. Zhongguo Lin Chuang Yao Li Xue Za Zhi.

[6] Shi Y, Pieniek M, Fard A, O’Brien J, Mannion JD, Zalewski A (1996). Adventitial remodeling after coronary arterial injury. Circulation.

[7] Herrmann J, Samee S, Chade A, Rodriguez Porcel M, Lerman LO, Lerman A (2005). Differential effect of experimental hypertension and hypercholesterolemia on adventitial remodeling. Arteroscler Thromb Vasc Biol.

[8] Glagov S, Weisenberg E, Zarins CK, Stankunavicius R, Kolettis GJ (1987). Compensatory enlargement of human atherosclerotic coronary arteries. N Engl J Med.

[9] Lafont AM, Chisolm GM, Wlaidow PL (1993). Postangioplasty restenosis in the atherosclerotic rabbit: Proliferative response or chronic constriction?. Circulation.

[10] Hong MK, Mintz GS, Abizaid AS (1999). Intravascular ultrasound assessment of the presence of vascular remodeling in diseased human saphenous vein bypass grafts. Am J Cardiol.

[11] Durand E, Addad F, Boulanger C (2001). Mechanical and functional predictive factors for restenosis and arterial remodeling after experimental angioplasty. Arch Mal Coeur Vaiss.

[12] Shi Y, O’Brien JE, Mannion JD (1997). Remodeling of autologous saphenous vein grafts. The role of perivascular myofibroblasts. Circulation.

[13] Berrutti L, Silverman JS (1996). Cardiac myxoma is rich in FXIIIa positive dendrophages: immunohistochemical study of four cases. Histopathology.

[14] Qiu ZB, Chen X, Wan S (2008). Phenotypic modulation of vascular smooth muscle cells and fibroblasts in vascular remodeling of pig vein grafts. Chinese Journal of Arteriosclerosis.

[15] Narvaez D, Kanitakis J, Faure M, Claudy A (1996). Immunohistochemical study of CD34-positive dendritic cells of human dermis. Am J Dermatopathol.

[16] Sun AJ, Cao PJ, Liu JJ (2004). Osteopontin enhance migratory ability of cultured aortic adventitial fibroblasts from spontaneously hypertensive rats. Sheng Li Xue Bao.

[17] Zhang DZ, Zhang TL, Hu CM, Liu QJ, Sun Y, Hao SH (2012). PTEN gene transfection on rabbit inhibition of restenosis experimental research. China Health and Nutrition.

[18] Stadius ML, Gown AM, Kernoff R, Collins CL (2010). Cell proliferation after balloon injury of iliac arteries in the cholesterol-fed New Zealand White rabbit. Atheroscler Thromb.

[19] Branca M, Ciotti M, Giorgi C (2007). Up-regulation of proliferating cell nuclear antigen (PCNA) is closely associated with high-risk human papillomavirus (HPV) and progression of cervical intraepithelial neoplasia (CIN) but does not predict disease outcome in cervical cancer. Eur J Obstet Gynecol Reprod Biol.

[20] Gingras M, Farand P, Safar ME, Plant GE (2009). Adventitia: the vital wall of conduit arteries. J Am Soc Hypertens.

[21] Ahmad S, Hewett PW, Al-Ani B (2011). Autocrine activity of soluble Flt-1 controls endothelial cell function and angiogenesis. Vasc Cell.

[22] Liu HW, Iwai M, Takeda-Matsubara Y (2002). Effect of estrogen and ATl receptor blocker on neointima formation. Hypertension.

[23] Stenmark KR, Gorasimovskaya E, Nemenoff RA, Das M (2002). Hypoxic activation of adventidal fibroblasts: role in vascular remodeling. Chest.

[24] Díez-Juan A, Andrés V (2003). Coordinate control of proliferation and migration by the p27Kipl/cyclin-dependent kinase/retinoblastoma pathway in vascular smooth muscle cells and fibroblasts. Circ Res.

